# Lessons learned from a full-scale bioterrorism exercise.

**DOI:** 10.3201/eid0606.000617

**Published:** 2000

**Authors:** R E Hoffman, J E Norton


### Lessons Learned from a Full-Scale Bioterrorism Exercise

During May 20-23, 2000, local, state, and federal officials, and the staff of three hospitals in metropolitan Denver, participated in a bioterrorism exercise called Operation Topoff. As a simulated bioterrorist attack unfolded, participants learned that a *Yersinia pestis* aerosol had been covertly released 3 days earlier at the city's center for the performing arts, leading to >2,000 cases of pneumonic plague, many deaths, and hundreds of secondary cases. The exercise provided an opportunity to practice working with an infectious agent and to address issues related to antimicrobial prophylaxis and infection control that would also be applicable to smallpox or pandemic influenza.

The sequence of events and the exact date of the exercise were not specified. However, the probable weekend and possible bioagents were suggested, which enabled us to begin preparations approximately 8 weeks ahead. Preparations included temporary appointments to the governor's 19-person Expert Emergency Epidemic Response Committee, which was created by enactment of a bioterrorism and pandemic influenza response law on March 15, 2000; recruitment of 25 epidemiologic and emergency management personnel from the 1,050 employees of our department and assignment to disaster response teams (e.g., surveillance, field investigation, and emergency management coordination); and establishment of a command center by reserving conference rooms and installing telephone, computer, and television equipment. Colorado's bioterrorism and pandemic influenza response law was not enacted to prepare for the exercise, but proved extremely useful. We recommend that state health agencies review their statutory authority and evaluate whether these laws would be adequate to deal with the threats of bioterrorism and pandemic influenza. During the exercise, we were provided information either from other participating agencies or from exercise controllers, and it was our task to investigate and respond. The staff reviewed mock medical records, analyzed laboratory specimens, interviewed patients, conducted meetings and group conference calls to assess surveillance data and decide on the next steps, drafted public health and executive orders, made written requests to federal officials for specific assistance, participated in news conferences, and packaged mock antibiotics for distribution at a prophylaxis clinic. By the end of day one, 783 cases and 123 deaths from plague had been reported from 16 hospitals (three participating hospitals and 13 simulated facilities). By the end of day two, 1,871 cases and 389 deaths were attributed to pneumonic plague, with 307 patients requiring ventilatory support. Cases were reported from six states outside Colorado. By the end of day three, 3,700 cases and 950 deaths were reported, including at least 780 secondary cases.

The exercise required state health department personnel to develop new working relationships. Although hospitals and local and state health agencies often collaborate with the Centers for Disease Control and Prevention in controlling an epidemic, we were unaccustomed to working closely with the Federal Bureau of Investigation, the U.S. Attorney for the District of Colorado, the Federal Emergency Management Agency, the Regional Office of the U.S. Public Health Service, and the Colorado Office of Emergency Management. Although lines of authority were clear, much time was spent in consultation and debate through scheduled bridge calls. Many persons joined these calls, and decision-making became inefficient, although not impossible. In a true incident, a central location for face-to-face meetings should be large enough to accommodate representatives from all agencies involved, but one difficulty encountered with arranging such meetings was that each agency seemed most comfortable in its own command center.

Another lesson we learned concerned our own organization. In addition to the surveillance, field investigation, and emergency management coordination teams, we needed teams to address laboratory testing, mass fatalities, legal problems, information technology, infection control, public and professional communications, and antibiotic and vaccine administration. During a disaster, no routine agency business can be conducted, as all employees are involved in the public health response. Finally, activities cannot depend on the direction of one or two key persons, such as the executive director and the state epidemiologist; other skilled, informed persons must be able to assume leadership roles. An electronic database documenting events, decisions, and requests for resources should be maintained. These logs enable staff to monitor the epidemic and the public health response rapidly.

In Colorado, where plague is endemic, we are familiar with the public health management of single plague cases, but the magnitude of the simulated epidemic and the fact that infection was spreading from person to person after a short (2- to 3-day) incubation period quickly overwhelmed the available resources. The challenge to our surveillance system was not in detecting the outbreak but rather in maintaining surveillance at each of the 22 acute-care hospitals in metropolitan Denver. Our hospital surveillance system usually relies on reporting by infection control practitioners, but during the exercise these practitioners had many additional responsibilities. In a true bioterrorist attack, emergency response teams of state or local health department employees should be set up and sent to each hospital to monitor cases and provide information to a central command center.

As more cases were identified, an anticipated issue emerged: who should receive antimicrobial prophylaxis? The governor's committee debated whether to limit prophylaxis to close contacts of infectious cases or offer it more widely (e.g., to all health-care workers, first responders, and public safety workers and their families) to gain the support and participation of key workers. The committee decided on the latter approach, but not unanimously.

The process of isolating plague patients until they are no longer contagious and identifying close contacts is typically straightforward. Isolation, however, was not possible during this exercise. The hospitals had too many patients and worried-well persons and too few health-care workers and empty rooms to permit isolation of pneumonic plague patients. Case reporting was delayed, and there were too few trained public health workers to conduct interviews and locate contacts in a timely manner. As a result, an executive order was issued quarantining all persons in metropolitan Denver in their homes. With infection control in the general population supposedly managed by the order, we could turn our attention to securing additional supplies, staff, beds, and equipment for the hospitals.

However, quarantining two million persons is not simple. Essential workers must be identified, be given prophylaxis and protective barriers, and be permitted to do their jobs. Other members of the community can stay in their homes only a few days before they need fresh supplies of food. Therefore, a one-time, blanket quarantine order is unlikely to be successful and cannot be enforced unless these and many other issues are addressed. The hospitals were quite demanding in their requests for reinforcements, and we made great efforts to assist them. However, by day three of the exercise it became clear that unless controlling the spread of the disease and triage and treatment of ill persons in hospitals receive equal effort, the demand for health-care services will not diminish. This was the single most important lesson we learned by participating in the exercise.

**Richard E. Hoffman and Jane E. Norton**
Colorado Department of Public Health and Environment, Denver, Colorado, USA

